# The Groundswell Community Surf Therapy Intervention for At-Risk Women and Changes in Body Acceptance, Resilience, and Emotional Regulation

**DOI:** 10.1177/27536130241278970

**Published:** 2024-08-30

**Authors:** Laura Franceschi, Natalie Small, Tamara Goldsby, Michael Goldsby, Shane Padamada, Michael G. Ziegler, Paul J Mills

**Affiliations:** 1 Groundswell Community Project, San Diego, CA, USA; 2 Center of Excellence for Research and Training in Integrative Health, 8784 University of California at San Diego, La Jolla, CA, USA; 3 Department of Medicine, 8784 University of California at San Diego, La Jolla, CA, USA

**Keywords:** surfing, at-risk women, resilience, emotional regulation, body acceptance

## Abstract

**Background:**

Surf therapy combines physical activity with social support to provide a healing environment.

**Objective:**

This exploratory pre-to post-intervention study examined the effects of a novel surf therapy program for women who experienced abuse, trauma, and/or mental illness on emotional regulation, resilience, body acceptance, and gratitude.

**Methods:**

Twenty-seven women (ages 25 to 54; mean 36.32 + SD 7.79) participated in an 8-week Groundswell Surf Therapy Program held in four different coastal cities in California. Standardized self-report questionnaires were administered prior to and following the therapy program, including the Body Acceptance Scale, the Connor-Davidson Resilience Scale (CD-RISC), the Affective Style Questionnaire, and the Gratitude Questionnaire-Six-Item Form (GQ-6) in a pre-post study design. Data were analyzed by repeated measures analysis of variance (ANOVA).

**Results:**

Body acceptance [*P* < 0.001; partial Eta squared = 0.472] and resilience were increased [*P* = 0.005; partial Eta squared = 0.319] following the surf therapy intervention. Emotional regulation was examined according to three subscales, with the adjust [*P* < 0.001; partial Eta squared = 0.397] and tolerate [*P* < 0.001; partial Eta squared = 0.299] subscales increasing following the intervention, and the conceal subscale [*P* = 0.459; partial Eta squared = 0.031] remaining unchanged. Gratitude scores were unchanged [*P* = 0.425; partial Eta squared = 0.026].

**Conclusion:**

A surf therapy program rooted in somatic and trauma-informed models was associated with improved resilience, emotional regulation, and body acceptance in at-risk women.

## Introduction

For millions of people around the globe, surfing is an integral part of life that supports their psychological health.^1^ As noted by Gibbs et al, “surf therapy provides a context for the experience of multiple determinants of wellbeing”,^2^ including physical exercise,^3,4^ positive emotions and creating,^5,6^ a connection with nature, and a community of surfers.^7^

Surf therapy programs for adults and children provide physical benefits, including helping with brain injury,^2^ post-hip surgery recovery,^8^ cerebral palsy,^9^ and Autism Spectrum and Down Syndrome^9^ symptomatology. Studies show that the mental and spiritual benefits of surfing include improved self-esteem, social connectedness, positive attitudes, and self-management in at-risk youth.^10-13^ Additionally, benefits of surfing for children and adolescents include reductions in total emotional and behavioral problems, as well as increases of youth pro-social behavior and quality of life.^14^

For individuals who have experienced significant trauma and/or abuse, there is often a need for alternative therapies that complement traditional psychotherapy,^15,16^ as traditional psychotherapy alone can show limitations such as moderate effect sizes as well as a lack of generalizability and endpoint improvement, depending on the target population.^17,18^ Such individuals are particularly vulnerable to mental health challenges^19,20^ and trust is a significant challenge for them.^21^ Thus for survivors of trauma, providing an emotionally safe alternative healing environment that includes feelings of comradery, friendship, and emotional support can be critical.^22^ Utilizing novel and/or alternative healing approaches with trauma survivors has shown encouraging results with regard to mental health symptom improvement,^23^ including such programs specifically designed for survivors of trauma such as domestic abuse and individuals with posttraumatic stress disorder (PTSD).^24,25^

Trauma survivors may too benefit from body-based (somatic) therapies such as surfing in attempting to enhance resilience.^26^ Female and male military service members and veterans demonstrate respite from (PTSD) symptoms utilizing surf therapy, including decreased depression and anxiety, as well as increased positive affect.^27,28^ Gerami, et al. studied the effects of a 6-week surf therapy on acute and chronic mental wellbeing in women and reported a strong positive relationship between surfing and improved mental wellbeing.^29^

There are other important domains relevant to women’s mental health that have yet to be examined in terms of surf therapy, including emotional regulation, body acceptance, and resilience.^30-32^ Emotional regulation, often called affective style, involves the ability to control or influence one’s emotions, particularly during times of stressful occurrences; psychologists are examining emotion regulation strategies as a subdiscipline.^33^ Emotion regulation plays an important role in developing and maintaining wellbeing.^30^ Specific affective style refers to a person’s ability to properly regulate their emotions to successfully adapt to situational demands, including avoiding unwanted and aversive emotional reactions.^30^

Building emotional resilience, defined as the ability to effectively cope with adversity,^34^ is a key feature of emotion regulation. Emotional resilience is viewed as a constructive approach to managing emotions and stress, and includes the ability to cope effectively with stressors and adversity and the ability to ‘bounce back’ from such adversity.^34^ Psychotherapy programs designed to promote emotional resilience show some success, including Cognitive Behavior Therapy (CBT) and Rational Emotive Therapy (RET).^35,36^ Exercise appears to afford a buffer to^37^ emotional stress and may enhance emotional resilience.^38^

Additionally, the literature demonstrates the importance of body acceptance in improved self-esteem, mental health^39^ and wellbeing.^40,41^ Emotional trauma survivors (including those with early childhood trauma) may be particularly vulnerable to having a negative body image as adults or adolescents.^42,43^ Thus, assistance in the enhancement of body image for trauma survivors would be of value. Positive body image is a relatively new and multi-faceted construct and includes body appreciation and acceptance.^44^ Body acceptance is the acceptance of one’s body even when one is not completely satisfied with all aspects of it.^45^ Greater body appreciation has been associated with psychological wellbeing.^40,41^ Exercise may enhance body acceptance, particularly in women as higher levels of body acceptance have been found among women who regularly exercise.^46^ Moreover, the type and manner of women’s conversations regarding exercise itself may lead to more body acceptance and appreciation levels.^47^

This exploratory intervention study sought to extend prior surf therapy studies by examining affective style, resilience, and body acceptance in women using a novel surf therapy program for at-risk women. The surf therapy curriculum is rooted in somatic, trauma-informed, and community therapy models to help women overcome psychological trauma and its mental health impacts.^26,48^

## Materials and Methods

### Intervention and Theoretical Underpinnings

The 8-week Groundswell Surf Therapy pre-post intervention design consisted of weekly two-hour sessions that included study participants, a Surf Therapy Facilitator, a Surf Safety Facilitator, and Volunteers. Groundswell’s goals include helping women build a connection to the ocean that supports resilience and confidence in everyday life. The nonprofit Groundswell Community Project facilitates surf therapy programs in California, Peru, Scotland, and England (https://www.groundswellcommunity.org/).

The Groundswell Surf therapy program has its roots in somatic and trauma-informed models to support the holistic healing of women.^26^ It is also informed by the field of Positive Psychology, including a focus on the client’s optimism, strength, and altruism.^49^ Additionally, the Groundswell program is based in Feminist Therapy Psychology, which is a person-centered, politically informed model that positions treatment within a cultural context.^50^ In the feminist model, as in the surf therapy program, healing is also undertaken in the community.^50^

The Groundswell surf therapy model believes feminist therapy allows and promotes:

Enrichment and absorption of positive experiences through experiencing and celebrating experiences with other women in the program; normalizing the trauma experience and recognizing that they are not alone; growing and expanding the surf therapy community connections outside of the surf therapy sessions; shifting from healing as the responsibility of the individual therapist to the community; and “positive peer pressure” (encouraging individuals to reach slightly beyond their usual comfort zone with new experiences that they might not otherwise have had the courage to experience alone).

The Groundswell Surf Therapy program also draws from the somatic therapies, eco therapy, and community therapy. Somatic therapists pursue a combination of psychotherapy and somatic treatments, with evidence that this combination may be more efficacious than either treatment alone in the treatment of mental health, including applying somatic therapies to a group setting.^51^ The Groundswell program utilizes a holistic somatic therapy approach in which trauma is viewed as not merely an event itself, but rather it is when the body experiences a disruption and overwhelm to the body and mind’s capacity to adapt, thrive, and flourish. Eco therapists embrace principles of Ecopsychology, with the belief that reconnecting with nature may improve physical and mental health and assist in emotional trauma while encouraging individuals to discover new solutions to long-standing problems.^52^ Finally, Community Psychology can be viewed as a form of positive psychology in which the goal is understanding the individual in the sociocultural context, including ethnicity.^53^ It integrates cultural, social, environmental, economic, political, and international effects on the individual to affect physical and mental health, empowerment, and positive change.

When considering the emotional trauma of participants, the Groundswell Surf Therapy model includes the following: “too much too soon” (stressors overwhelm the individual early in life); “too much for too long” (stressors overwhelm the individual for an extended period of time); “not enough for too long” (lack of emotional support and bonding for an extended period of time); power and agency have been taken away from the person; the stressors outweigh the resources available to navigate the stressors; when the individual’s primal protective instincts, intuitions, and responses have been thwarted; and when there is not sufficient time, space, or permission to emotionally heal.

### Groundswell Curriculum Format

Each surf therapy session builds on the prior and covers mental health topics regarding belonging, fear, negative self-talk, body relationship, emotional regulation, resilience, self-awareness, and interpersonal relationships with others and nature. The model holds a strong belief that human mental, physical, and spiritual health are directly correlated with the health of the earth and sea. Ocean care and self-care practices are interrelated, explored, and practiced in this program.

Groundswell Surf Therapy sessions had specific surf goals and therapy goals and utilized the following curriculum and intervention format (see Appendix 1 and Appendix 2). Surf therapy sessions over the 8-weeks were 90 to 120 minutes in duration. The first 30 minutes to 1 hour of the surf therapy session occurred on land in which participants took part in a group therapy circle, a one-on-one conversation with a surf therapy staff member, and establishing a new surf goal for the week. During the first session, participants learned water safety protocols, as well as the Groundswell Curriculum to build their personal relationship and safe access to the ocean. Study participants had varied surf experience from no ocean and/or no swimming experience to some prior surfing experience, including a prior traumatic event while surfing that stopped them from continuing with their surf practice.

Session formats were as follows:

Gear Set Up. Setting the physical space to hold the psychological safe space.

Warm Up. Briefing with Facilitators and Volunteer staff.

Welcome and Check-in with Participants.

• Grounding and breathing exercises.
• “We Voice”: Grounding and connecting to the body. Connecting breath and body.
• “I Voice”: “I am ____” exercise.
• Land session: psychoeducation to prepare for practice in the sea.
• Partner Share: Sharing with another participant their fears and hope about the session.

Waves.

• Women in Waves: Time in ocean and learning to surf.
• Wisdom from the Waves (I Voice): reflecting on experience in the water and participants sharing experience.
• We Voice: Breathing and grounding.

Cool Down and Closing.

• Closing the session, bonding, practicing gratitude.
• Check out: homework, surf journal. One-to-one check out with a facilitator.

The Groundswell Facilitators ensured study participants felt safe in the water and introduced surfing in the second and third sessions. Each participant was assisted by both the Surf Safety Facilitator and a trained Volunteer. Volunteers provided both instruction and physical assistance. Following the surf sessions, study participants met back on land and described how they felt after their time in the water and what they learned in the ocean.

### Study Participants

Thirty-one women trauma survivors enrolled in Groundswell Surf Therapy programs and served as study participants. Participants were recruited from four different Groundswell intervention sites, in San Diego, Oceanside, Los Angeles, and San Francisco, CA and took part in the surf therapy program in groups of 8 to 10 participants. Potential study participants were told that the purpose of the study was to examine the program’s effect on mood and quality of life in women who report abuse, trauma, and/or mental health illness. Participants were female, eighteen years of age or older and had experienced emotional trauma.

### Inclusion and Exclusion Criteria

In order to participate in the study, women could not be in an inpatient psychiatric facility. The following was determined by Groundswell staff via initial questionnaire and subsequently meeting individually with each potential participant: Participants needed to be functioning at a level in which they could arrive at surf therapy sessions independently (without surf therapy program staff transportation). Additionally, participants needed to be in possession of their own electronic devices (phone, tablet, or computer) in order to complete the necessary online paperwork and be able to communicate via phone and email to receive information regarding program times, location, and details. The exception to these criteria was for those participants who came to the surf therapy program through a partnering organization that provided these services for the individual. Additionally, the Groundswell surf therapy program is not an in-patient or long-term clinical support program, thus clients needed to have such clinical support systems in place where needed.

Approximately 50% of potential participants were referred to the program from therapists and community health workers and the remaining 50% were through word-of-mouth referral. Participant traumatic event(s) included divorce, domestic abuse, relocation, loss of loved one, job or home, illness diagnosis, racism and violence, major life changes, attempting to leave sex trafficking, homelessness, and/or being a refugee. Mental health impacts included trauma, addiction, abuse, eating disorders, anxiety, extreme lack of self-confidence, lack of community, suicidal ideation, and mental health disorders. Thus, the surf therapy program had individuals coming together with a variety of mental health issues.

### Staff Training

Surf Therapy Facilitators were certified using the Groundswell Facilitator curriculum. Surf Safety Facilitators were individuals with a developed surf practice and experience in surf coaching. They held first aid and water safety certifications and completed the SALT ocean safety training provided by the Huntington Beach lifeguards (https://surfcitybreak.com/salt-water-training-classes/?utm_source=rss&utm_medium=rss&utm_campaign=salt-water-training-classes). Surf Therapy Facilitators, as well as Surf Safety Facilitators, completed the Groundswell Community project’s 8-week Surf Therapy for Trauma Recovery training. This included Groundswell materials and Embodied Feminine leadership model training by Shakti Rising facilitators who had weekly clinical supervision to support them in facilitating the programs.

The Surf Program Volunteers attended Groundswell’s specialized Volunteer training, including trauma informed training (https://www.groundswellcommunity.org/groundswell-volunteer) and had CPR and First Aid certifications.

Staff facilitators attended trainings, followed the curriculum, had specified roles, and maintained specified protocols set out by the Groundswell surf therapy program.

### Institutional Review Board Approval

The study was approved by the U.C. San Diego Institutional Review Board (#190586). Written informed consent was obtained from each participant before enrolling in the study.

### Data Collection and Study Questionnaires

Study participants completed the program questionnaires (pre- and post-participation in the surf therapy program) at their residences, utilizing their own electronic devices.

#### Body Appreciation Scale (BAS-2)

A 10-item self-report scale that measures positive body image^54,55^ and is the refined version of the original 13-item BAS scale.^56^ The BAS-2 is on a 5-point Likert scale from 1 – 5 (1 = never and 5 = always). Thus, higher scores on the scale represent positive body appreciation. Tylka and Wood-Barcalow (2015) conducted three studies on the BAS-2, with confirmatory factor analysis supporting the unidimensionality and invariance across sex and sample type.^44^ The BAS-2’s reliability indices (internal consistency and 3-week stability), unidimensional factor structure and validity estimates (i.e., construct, criterion, discriminant, and incremental) were upheld for participants. In each of the three studies, internal consistency was supported, with Cronbach’s α ranging from .77 to .97. In this study sample the Cronbach’s α was 0.83.

#### The Connor-Davidson Resilience Scale (CD-RISC-10)

A 10-item unidimensional self-report scale measuring resilience^57,58^ and the ability to deal effectively with trauma, stressful events, and tragedy. It is comprised of 10 of the original 25 items, with the possible points ranging from 0-40. The measure is on a 0-4 Likert Scale with 0 representing “not true at all” and 4 representing “true nearly all the time.” Thus, high scores on this scale indicate a high level of ability to bounce back or recover from stressful life events and tragedy.

A study of the psychometric properties of the CD-RISC-10 in the general population and patient samples revealed adequate internal consistency, test-retest reliability, and convergent and divergent validity.^59^ Cronbach’s α for the full scale was found by Connor and Davidson^57^ to be 0.89 and item-total correlations ranged from 0.30 to 0.70. In a study conducted by Gonzalez and colleagues,^60^ researchers concluded that the 10-item scale was found to be psychometrically superior to the 25-item scale and the 5-item scale. This conclusion was reached due to results from item-level analyses and confirmatory factor analyses. Additionally, Gonzalez and colleagues found that the 10-item scale correlated positively and moderately with positive affect, using structural equation modeling. Further, this scale correlated negatively with performance anxiety and negative affect, establishing convergent and divergent validity. Additionally, a study conducted using the 10-item scale examining resilience in adult women demonstrated high internal consistency.^59^ In this sample the Cronbach’s α was 0.83.

#### The Affective Style Questionnaire

A 20-item self-report scale which yields three subscales: conceal, adjust, and tolerate.^61^ Studies indicate that lower scores for the adjusting subscale are found in individuals suffering from affective disorders as compared to individuals suffering from anxiety, and that the adjusting and concealing subscales are important indicators for depression, anxiety, and stress among clinical populations.^30^ The ASQ is on a Likert-type scale from 1-5, with 1 representing “not true of me at all” and 5 representing “extremely true of me”. Factor analysis revealed three factors: Concealing subscale (habitual attempts to conceal or suppress affect; 8 items); Adjusting subscale (a general ability to manage, adjust, and work with emotions as needed; 7 items); and Tolerating subscale (an accepting and tolerant attitude toward emotions; 5 items).^61^ In our sample, the ASQ demonstrated satisfactory internal consistency: *Concealing* (Cronbach’s α = .78), *Adjusting* (α = .75), and *Tolerating* (α = .67) subscales.

#### The Gratitude Questionnaire-six-item Form (GQ-6)

A six-item self-report questionnaire designed to assess individual differences in the proneness to experience gratitude in daily life.^62^ We and others have previously shown relationships between higher trait gratitude and mental health and well-being.^63,64^ Given those findings, coupled with studies showing an association of gratitude with well-being in at-risk women,^65^ we included the GQ-6 to examine if the surfing intervention might increase trait gratitude. The GQ-6 measure is on a Likert-type scale of 1-7, with 1 representing “strongly disagree” and 7 representing “strongly agree”. It has demonstrated a robust one-factor solution with adult populations and strong internal consistency, with a Cronbach alpha of .82^62^ and Cronbach α of 0.92 in a cardiac population.^63^ In this sample the Cronbach’s α was 0.63.

### Data Analysis

Data were initially examined using the SPSS descriptive statistics function which provides means, standard deviations, skewness, and kurtosis (SPSS version 29). These statistics were in the acceptable normal ranges for proceeding with the parametric analyses. Each variable was tested via separate one-way repeated measures analysis of variance (ANOVA).

## Results

Twenty-seven women trauma survivors (ages 25 to 54; mean 36.32 + SD 7.79 completed the pre and post questionnaires: 6 Asian, 12 Caucasian, 7 Latina, 1 Native American, 1 East Indian). Means + SD are reported.

Body acceptance scores increased significantly following the surf therapy intervention, 34.22 (9.31) to 40.07 (6.77) (F1,26 = 0.658, *P* < 0.001) (n = 27) (Table 1). Similarly, resilience scores increased significantly following the surf therapy intervention, 26.29 (7.33) to 30.48 (6.15) (F1,26 = 9.57, *P* = 0.005) (Table 1).

**Table 1.** Study Variables and Statistical Outcomes (Means + SD).

	Pre-Surf Therapy	Post-Surf Therapy	Partial Eta Squared	*P* value
Body acceptance scale (n = 27)	34.22 (9.31)	40.07 (6.77)	0.472	<0.001
Connor-davidson resilience scale (n = 27)	26.29 (7.33)	30.48 (6.15)	0.319	0.005
Affective style questionnaire				
Conceal	24.40 (5.10)	23.77 (4.96)	0.031	0.459
Adjust	19.22 (4.54)	22.11 (3.90)	0.397	<0.001
Tolerate (n = 27)	16.66 (4.65)	18.48 (3.51)	0.299	<0.001
Gratitude questionnaire-six-item form (n = 27)	27.29 (3.40)	27.88 (3.01)	0.026	0.425

Affective style was examined according to three subscales.^61^ The Conceal subscale was unchanged following surf therapy, 24.40 (5.10) to 23.77 (4.96) (F_1,26_ = 0.565, *P* = 0.459), whereas the Adjust, 19.22 (4.54) to 22.11 (3.90) (F_1,26_ = 14.85, *P* < 0.001) and Tolerate, 16.66 (4.65) to 18.48 (3.51) (F1,26 = 12.66, *P* < 0.001) subscales were significantly increased (n = 27) (Table 1).

Gratitude scores were unchanged following surf therapy, 27.29 (3.40) to 27.88 (3.01) (F1,26 = 0.658, *P* = 0.425) (n = 27).

Across all study sites, the average attendance rate for the 8-week program was 86.07%.

## Discussion

This study examined the effects of a novel surf therapy program on aspects of wellbeing in women recovering from trauma. Surfing “is a profoundly meaningful practice that brings physical, psychological, and spiritual benefits”.^1^ Studies seeking to identify theoretical mediators to understand how surfing supports health and wellbeing include prominent theories such as mastery, respite, and social connectedness, as well as theories fostering personal growth, the development of healthy relationships, improved self-management, and the creation of emotional and physical safe spaces.^6,7,10^

In the present study, several scales measuring the ability to effectively deal with emotional adversity without being overwhelmed by one’s emotions were examined and found to be significantly increased following the surf therapy intervention. Emotional resilience may be thought of as the ability to bounce back after trauma or adversity. Resilience can be considered an important aspect of wellbeing and effective functioning in day-to-day life, and is particularly important for those individuals who have experienced trauma.^66,67^ Thus, for the present study’s participants who had experienced trauma, it is an especially important finding that their resilience scores on *The Connor-Davidson Resilience Scale* had significantly improved following the surfing intervention. Our study findings regarding resilience are consistent with a prior 8-week surf therapy study and a prior 6-week surf therapy study that examined effects on resilience in adolescents.^10,11^

Similar to emotional resilience, the Tolerating subscale of *The Affective Style Questionnaire* examines the individual’s ability to experience and manage one’s arousing emotions, especially in the face of adversity. The current study found a significant increase in the Tolerating subscale, which has also been supported in a previous surf therapy program.^10^ Closely aligned with emotional resilience and the Tolerating subscale, the Adjusting subscale of the *Affective Style Questionnaire* measures the ability to adjust, work with, and manage one’s emotions.^61^ The present surf therapy intervention showed a significant increase in the Adjusting subscale following the surf intervention. Thus, emotional resilience and related concepts significantly increased with the present surf therapy intervention. Given the significant increase in emotional resilience scores, this type of surf therapy may be a promising approach to begin emotional healing in at-risk women for mental health disorders, especially those with a history of trauma, abuse, or neglect.

The current study also examined the relationship between surf therapy and body acceptance which has not previously been studied in relation to surf therapy in published literature. The finding that body acceptance was significantly improved on the *Body Appreciation Scale* in response to the surfing intervention is aligned with other research involving outdoor activities. In general, exposure to activities in natural environments and outdoor activities is linked with an improved body image, a concept aligned with body acceptance.^68^ Exercise alone too, has been associated with greater body acceptance in women.^46^ Moreover, social support has been found to have an impact on body image and acceptance in women in a variety of contexts,^69,70^ including the involvement of women in physical activities.^71^ One study found that in a 6-week adventure program for young adults with cancer, that included surfing, body image was improved the first time they participated in the program, compared to a wait-list control group.^72^

Based on prior research on gratitude and well-being,^63^ including in at-risk women,^65^ we examined the potential effects of the surf therapy program on trait gratitude. Gratitude scores did not significantly change. Prior studies using the GQ-6 found that individuals with higher state gratitude scores were more trusting.^73^ Individuals who have difficulties with trust, such as traumatized individuals,^74^ may not readily have improvements in gratitude. Confino and colleagues (2023) distinguish between state and trait gratitude in the following manner: “state gratitude” relates to an affective-cognitive state and attribution-dependent short-term state, whereas “trait gratitude” represents a longer-term broader life orientation.^75^ As the GQ-6 measures trait gratitude, perhaps a longer-term surf intervention would be needed to observe possible effects on trait gratitude. Additionally, for future research it may be useful to include a measure of state gratitude such as the State Gratitude Scale.^76^ Moreover, a relationship has been demonstrated between gratitude and social support.^77^ In our study, we did not assess participant’s home or social support system (or lack thereof). It is possible that for participants lacking a strong social support system, this lack may have contributed to a lack of change in gratitude scores.

We acknowledge limitations of this study, including that we did not have a control group or another independent variable. We also acknowledge the study’s modest sample size. We did, however, observe positive changes for body acceptance, resilience, and the adjust and tolerate subscales of emotional resilience, while not observing changes in the conceal subscale of emotional resilience and gratitude, suggesting perhaps a specific rather than a generalized expectation response. While this study examined a surfing intervention, it may be that overcoming any physical challenge in nature would provide similar benefits.

## Conclusion

Participants in this surf therapy intervention study showed improved resilience and emotional regulation, as well as body acceptance. In addition, the program’s strong attendance rates suggest it had high perceived value among the women participating. Future studies might compare the results of the Groundswell intervention to other alternative therapies, both indoor and in nature. For example, it would be especially relevant to examine the effects of surf therapy to other outdoor programs, including forest therapy (walking in a forest, which has been referred to as “forest bathing”) or more active outdoor activities such as kayaking or open-water swimming in seas or lakes. This would provide an indication if surfing specifically is therapeutic or if it is rather outdoor group activities per se that provide such benefits for individuals who have experienced trauma. Other possible group comparisons could involve more meditative approaches such as yoga or meditation, either indoor or outdoor. An additional potential comparison group might include a wellness-education group to determine if obtaining knowledge in mental health and emotional healing techniques alone would provide substantial benefit to such vulnerable populations. Such future studies would be informative and help us understand how these findings might generalize to other intervention approaches and populations.

## Appendix 1 Groundswell Healing Themes

Themes covered in each Groundswell Surf Therapy Session:

• Connecting breath to movement and breath as a regulator
• All of our emotions are important, and like Mother Ocean, we hold space for all emotions
• Celebrating and holding space for your own unique expression
• Waves of love: holding space to honor each voice
• Welcome the body to the healing journey: Embodying emotions
• Healing practice
• Play & failure are essential parts of the healing journey
• Power of healing within community: *You are not alone*
• We each have our own unique journeys that have brought us here. Each person’s experience is uniquely their own
• Mother Ocean as a guide for healing and a mirror for ourselves

## Appendix 2 Description of Surf Sessions

### Session 1: Welcome to Your Surfing to Self-Love Practice

Surf Goal: Build a relationship with Mother Ocean through learning how to be safe

Therapy Goal: Setting intentions and embodying your strength (Power stance and empathy practice)

### Session 2: Welcome to Play

Surf Goal: Building knowledge of Mother ocean’s Movement and waves through body boarding

Therapy goal: Welcoming play and joy into healing (sea animal embodiment and ocean play)

### Session 3: Welcome to Your Body

Surf Goal: Surf Pop-up

Therapy Goal: What tools does your body already have for emotional regulation, surviving, and thriving -- (Breath to pop-up)

### Session 4: Welcome to Mother Ocean’s Body

Surf Goal: How to read the waves & move through them

Therapy Goal: playing with emotions as waves and conscious choosing how we move through them - (Wave Emotions Meditation + Wave Drama + Ocean Diagram)

### Session 5: Welcome to Your Waves

Surf Goal: Surf Etiquette

Therapy Goal: You belong (Agreements practice: burning old to welcome in new)

### Session 6: Welcome to Falling Forward

Surf Goal: Practicing your surf practice and how falling is key to success

Therapy Goal: Building new relationship with failure (You make the world more beautiful flowers ceremony)

### Session 7: Welcome to Sea-Love & Self Love

Surf Goal: Ocean conservation

Therapy Goal: Self-love = Sea Love (Plastic confessionals + move from guilt and shame to action and community)

### Session 8: Welcome to Surf Sisterhood

Surf Goal: Sustainable surf practice for Self-Love, Sea Love, and Sisterhood

Therapy Goal: Sustainable healing journey within community (Blue mind marble bracelet)
